# Rodent-borne infections in rural Ghanaian farming communities

**DOI:** 10.1371/journal.pone.0215224

**Published:** 2019-04-24

**Authors:** Shirley C. Nimo-Paintsil, Elisabeth Fichet-Calvet, Benny Borremans, Andrew G. Letizia, Emad Mohareb, Joseph H. K. Bonney, Kwasi Obiri-Danso, William K. Ampofo, Randal J. Schoepp, Karl C. Kronmann

**Affiliations:** 1 United States Naval Medical Research Unit Number 3, Ghana Detachment, Accra, Ghana; 2 Department of Virology, Noguchi Memorial Institute for Medical Research, Legon, Accra, Ghana; 3 Department of Virology, Bernhard-Nocht Institute of Tropical Medicine, Hamburg, Germany; 4 Department of Ecology & Evolutionary Biology, University of California, Los Angeles, California, United States of America; 5 Hasselt University, Hasselt, Belgium; 6 Department of Virology, United States Naval Medical Research Unit No. 3, Cairo, Egypt; 7 Kwame Nkrumah University of Science and Technology, Kumasi, Ghana; 8 Diagnostic Systems Division, United States Army Medical Research Institute of Infectious Diseases, Frederick, Maryland, United States of America; 9 Department of Internal Medicine, Naval Medical Center, Portsmouth, Virginia, United States of America; Tulane University, UNITED STATES

## Abstract

Rodents serve as reservoirs and/or vectors for several human infections of high morbidity and mortality in the tropics. Population growth and demographic shifts over the years have increased contact with these mammals, thereby increasing opportunities for disease transmission. In Africa, the burden of rodent-borne diseases is not well described. To investigate human seroprevalence of selected rodent-borne pathogens, sera from 657 healthy adults in ten rural communities in Ghana were analyzed. An in-house enzyme-linked immunosorbent assay (ELISA), for immunoglobulin G (IgG) antibodies to Lassa virus was positive in 34 (5%) of the human samples. Using commercial kits, antibodies to hantavirus serotypes, Puumala and Dobrava, and *Leptospira* bacteria were detected in 11%, 12% and 21% of the human samples, respectively. Forty percent of residents in rural farming communities in Ghana have measurable antibodies to at least one of the rodent-borne pathogens tested, including antibodies to viral hemorrhagic fever viruses. The high seroprevalence found in rural Ghana to rodent-borne pathogens associated with both sporadic cases and larger disease outbreaks will help define disease threats and inform public health policy to reduce disease burden in underserved populations and deter larger outbreaks.

## Introduction

Rodents are reservoirs and/or vectors for several pathogens known to cause disease in humans. Disease may occur as sporadic individual cases as well as larger outbreaks [1–3]. Rodents are the reservoir of viruses in the Bunyaviridae and Arenaviridae families responsible for viral hemorrhagic fevers, including hemorrhagic fever with renal syndrome (HFRS), hantavirus pulmonary syndrome (HPS) and Lassa fever (LF). In 2016, the latter was included in the initial list of eight severe emerging diseases with potential to generate a public health emergency of international concern and identified by the World Health Organization for urgent research and development [4].

Pathogens, such as hantavirus and Lassa virus (LASV), have limited reservoir host species. Three different species of rodent have recently been shown to harbor LASV namely; *Mastomys natalensis*, as the primary host, along with *Mastomys erythroleucus* and *Hylomyscus pamfi* [5]. Lassa virus infects multimammate mice, *M*. *natalensis* and *M*. *erythroleucus*, which are abundant in many regions throughout Sub-Saharan Africa, and nosocomial outbreaks of LF in humans have occurred in some West African countries [6]. Two discontiguous areas in West Africa are the best known areas of LF transmission—Nigeria to the east of Ghana and Sierra Leone to the west of Ghana. During the current LF outbreak in Nigeria, cases have also been reported in Ghana, though the relation to the Nigerian outbreak is unclear, as is the presence of unrecognized, autochthonous transmission, if any outside of the outbreak [3]. Ecological niche modelling has over the years identified key environmental variables correlated with LF outbreaks, particularly rainfall, human population density and farm fields [7, 8]. Warmer temperatures and increased rainfall are future climate projections for West Africa, and are predicted to increase the climatic suitability for *M*. *natalensis* across much of the region. High prevalence of human LASV antibodies has been reported in West Africa, particularly in Guinea, Sierra Leone, Liberia and Nigeria [6]. There are limited data from other West African countries, but the emergence of new cases in Togo and Benin in 2014 and 2016, respectively confirm the circulation of LASV in these areas [6, 9, 10]. In Ghana, a study of rodents in the same villages as this present report did not find evidence of LASV in *Mastomys* species, although one Lassa-like virus was detected in *Nannomys baoulei* [11].

Hantaviruses have been discovered in rodents and other small mammals, such as shrews and moles [12]. In Africa, serological indications of human hantavirus infections were detected in 6 central African countries, with an overall seroprevalence of 6% [13]. Other serological surveys have suggested infections in Senegal, Nigeria, and Egypt [14–16]. Some studies in Guinea found the prevalence of hantavirus antibodies in the local population to be 1.2%, while seroprevalence of specific neutralizing antibodies against Sangassou virus (the first described indigenous African hantavirus) was 4.4% [17].

*Leptospira* species infect a wide range of hosts, including large mammals, reptiles, birds, and amphibians, although rodents are the most important maintenance host [18]. Outbreaks of the disease are reported frequently, typically after floods [19–21]. Leptospirosis, like hantavirus infections, has not been widely studied in Africa. In a human serologic survey undertaken in Egypt, 5.6% of the 513 humans showed leptospiral microscopic agglutination titres of 1:128 and above [22]. In the arid Mogadishu area of Somalia a serosurvey showed 37% seropositivity for *Leptospira* [23]. Macro-agglutination tests on slides for detecting anti-*Leptospira* antibodies were carried out on 235 sera in Gabon of which 37 (15.7%) were positive [24].

There is little knowledge about rodent-borne diseases in Ghana and their impact on public health decisions to prevent disease and/or manage outbreaks. Studies in neighboring countries provide information on the rodent-borne infections under investigation, but little is known about the rate for human infection from these pathogens in Ghana. We therefore conducted a survey across the country to investigate seroprevalence rates of rodent-borne diseases in the human population. Ghana is situated directly between two well-known LASV endemic areas, Sierra Leone and Nigeria, and both the rodent reservoirs and a suitable environment are present. To examine the possibility of unrecognized LASV circulation in Ghana as well as outbreak potential for other rodent-borne diseases, we assessed seroprevalence of antibodies against LASV, hantavirus and *Leptospira*. Possible correlations between rodent populations, pathogens, environmental factors and human seroprevalence for these zoonotic infections were also examined.

## Materials and methods

### Field procedures

#### Study sites

Noguchi Memorial Institute for Medical Research, University of Ghana, Legon, Ghana (Certified Protocol Number: 013/09-10). United States Naval Medical Research Unit No. 3, Cairo, Egypt (IRB No. N3 1008; DoD No. NAMRU3.2010.0008). Written form of consent obtained.

**Fig 1:**
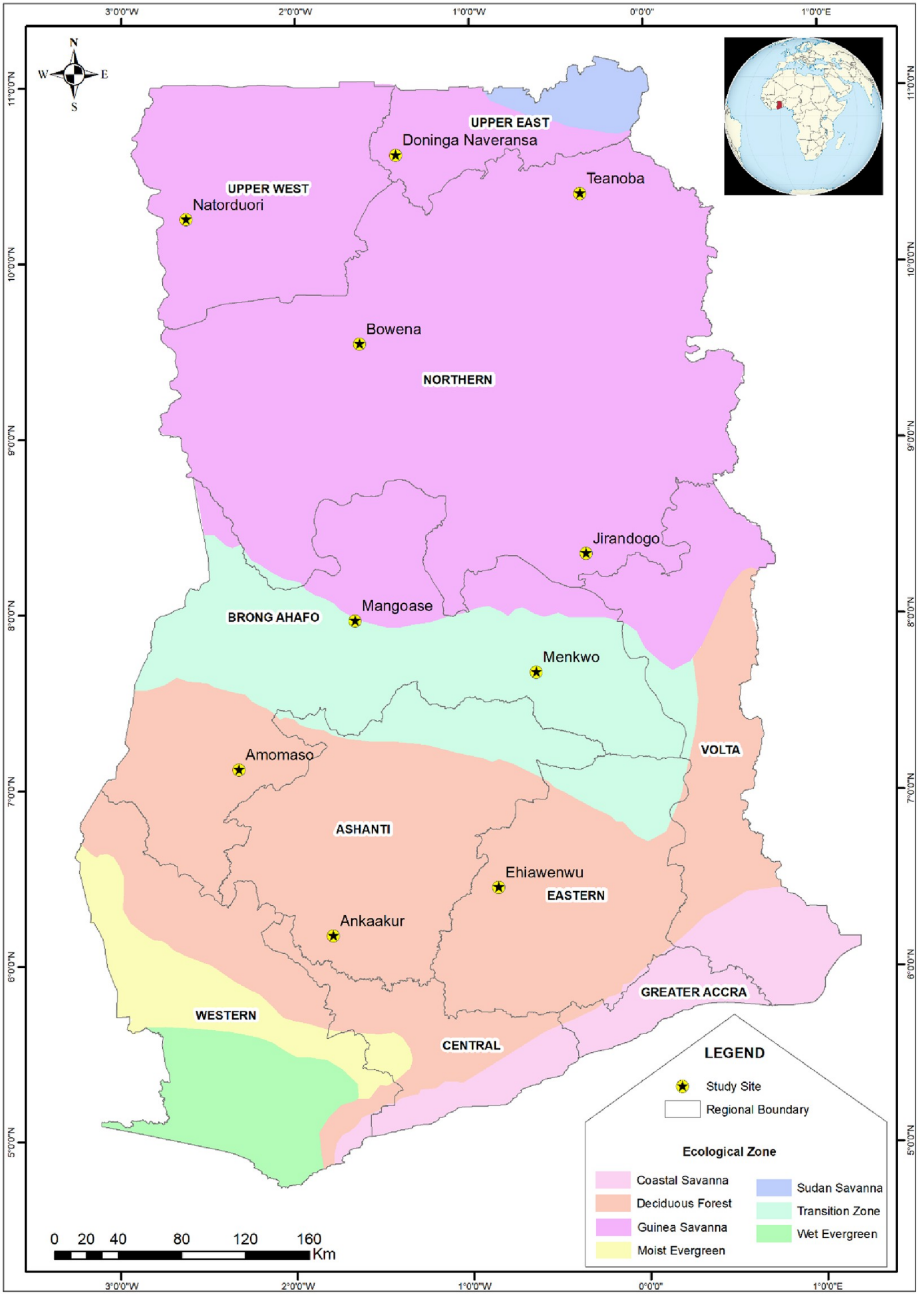
Ecological zones of Ghana showing study sites. (Ankaakur, N = 31; Ehiawenwu, N = 67; Amomaso, N = 70; Menkwo, N = 70, Mangoase, N = 69; Jirandogo, N = 70; Bowena, N = 70; Natorduori, N = 70; Teanoba, N = 70; Doniga Naveransa, N = 70).

Ten villages were selected as described previously to represent seven northern villages (guinea savannah woodland) most likely to have LASV, along with three lower risk (transition zone) villages (Fig 1) [11]. Criteria targeted remote, rural villages with population sizes between 500 and 2,000 people, located at least 20 km from major roads or urban centers, which are favorable environments for rodent species such as *Mastomys natalensis* [25]. At each of the ten villages, both rodent sampling and human blood draws were done concurrently during the rainy seasons between August 2010 and August 2011. Findings of the rodent sampling have been described by Kronmann et al., 2013.

#### Human subject enrollment

Blood samples were obtained by venipuncture from 657 healthy individuals (≥ 18 years) after informed consent was obtained. A convenience sample was taken by enrolling volunteers from a central location within each village. Serum was separated from approximately 5 mL of blood by centrifugation on site, stored in a tank with liquid nitrogen and transported to the laboratory at Noguchi Memorial Institute for Medical Research (NMIMR) where all laboratory investigations were carried out. Questionnaires were also administered to consented individuals to obtain data on demographics and possible risk factors.

#### Ethical statement

A public forum for each village was held on day of entry in consultation with leaders from the local community to describe the study. All volunteers were enrolled at a central location in subsequent days after they had consented. Ethical clearance was given by the Institutional Review Boards of Noguchi Memorial Institute for Medical Research (NMIMR), and the United States Naval Medical Research Unit No. 3, Cairo, Egypt (NAMRU-3) in compliance with all applicable federal regulations governing the protection of human subjects.

#### Questionnaires

Participants were asked questions regarding their housing, occupation, and food storage practices. As a measure of exposure to rodents, enrolled subjects were asked to describe how frequently they had seen rodents in and around their houses (never, sometimes or most days). Clinical data was also assessed including fever within the past year, hospital admissions and hearing loss or changes (S1 File).

### Laboratory procedures

#### Serology

An in-house enzyme-linked immunosorbent assay (ELISA) from the United States Army Medical Research Institute of Infectious Diseases (USAMRIID) was used to detect anti-LASV antibodies. Immobilized antigen was isolated from Vero cells infected with LASV and microtiter plates were coated with LASV positive (+) and negative (-) cell slurry at 1:1000 dilution. Serum was considered positive for anti-LASV IgG when the optical density (OD) was greater than 0.2 over background [determined by subtraction of the average of OD values of the LASV (+) and (-) slurry of the same sample]. The ELISA positive samples were further tested with a plaque reduction neutralization test (PRNT) for Josiah strain LASV at USAMRIID. The sensitivity of the assay detects 10^6 pfu/ml for LASV Josiah (Lineage 4). The capture antibody is a rabbit polyclonal, therefore, it picks up all Lineages 1, 2, 3, 4, and 5 depending on the relatedness of the viruses [26].

Antibodies against Dobrava/Hantaan and Puumala hantaviruses were detected by ELISA using commercial kits from Progen BioTechnik GmbH, Germany. A ratio of the absorbance of a patient’s sample to the reference control of greater than 1.5 indicated detection of IgG antibodies against Puumala or Dobrava/Hantaan.

A Serion ELISA classic *Leptospira* IgG kit (Serion GmbH, Germany) was used to screen sera for anti-*Leptospira* antibodies. This sensitive quantitative test detects genus-specific human antibodies against *Leptospira* in serum or plasma; concentrations higher than 9 U/ml indicated *Leptospira* exposure.

#### Data analysis

Data were collected and analyzed with the statistical package for social sciences (SPSS) software version 20, R statistical software and R package lme4 [27,28]. Associations between antibody seroprevalence and other variables (such as ecozones, sex, age, housing roof or wall type, village and rodent observation) were analyzed using generalized linear mixed models, assuming a binomial error distribution and logit link function. Where necessary, effects of village and ecozone were accounted for as random effects. Likelihood ratio testing (assuming a Chi-squared distribution) of hierarchical subsets was used to assess statistical significance of model variables.

## Results

### Demography, domestic habitat and rodent abundance

A total of 657 human subjects between 18 and 98 years of age (mean age 41.4 ± 18.3 years standard deviation) were enrolled in the study from ten villages. Table 1 shows the demographics of the study population. Using exact binomial test, there were more females (356) than males (297), (P = 0.035, 95% CI 50.3%-58.0%). Most participants lived in mud-walled huts with aluminum roofs with typical occupancy of 6–10 persons, without either electricity (92%) or indoor plumbing (89%). Popular food storage places observed were room in house (52%), kitchen (24%) and separate building (15%). Food stuff were sometimes stored in baskets, sacks and on concrete floor. Sixty-five percent (429) of the study population reported seeing rodents in and around their home most days, thirty percent (197) observe rodents sometimes whereas four percent (23) reported never seeing rodents. Seventy-one percent (486) reported that they worked as farmers. See S1 Table, for demographic information of the different villages.

**Table 1:** Demographic information.

Demographic		Number (percentage)
Gender	Male	297 (45%)
	Female	356 (54%)
	Missing gender	4 (1%)
Age	18-25yrs	152 (23%)
	26-35yrs	152 (23%)
	36-45yrs	137 (21%)
	46-55yrs	72 (11%)
	56-65yrs	55 (8%)
	Above 65yrs	86 (13%)
	Missing age	3 (0%)
Housing, occupancy	1–5 persons	152 (23%)
	6–10 persons	288 (44%)
	Greater than 10 persons	208 (32%)
	Missing data	9 (1%)
Housing, wall type	Block or brick	103 (16%)
	Mud	521 (79%)
	Thatch	26 (4%)
	Missing data	7 (1%)
Housing, roof type	Aluminum	452 (69%)
	Straw, Thatch or Bamboo	193 (29%)
	Missing data	12 (2%)
Housing, indoor plumbing	Yes	66 (10%)
	No	584 (89%)
	Missing data	7 (1%)
Housing, electricity	Yes	39 (6%)
	No	602 (92%)
	Missing data	16 (2%)
Rodent observation	Most days	429 (65%)
	Sometimes	197 (30%)
	Never	23 (4%)
	Missing data	8 (1%)

### Lassa virus immunoglobulin G

**Fig 2:**
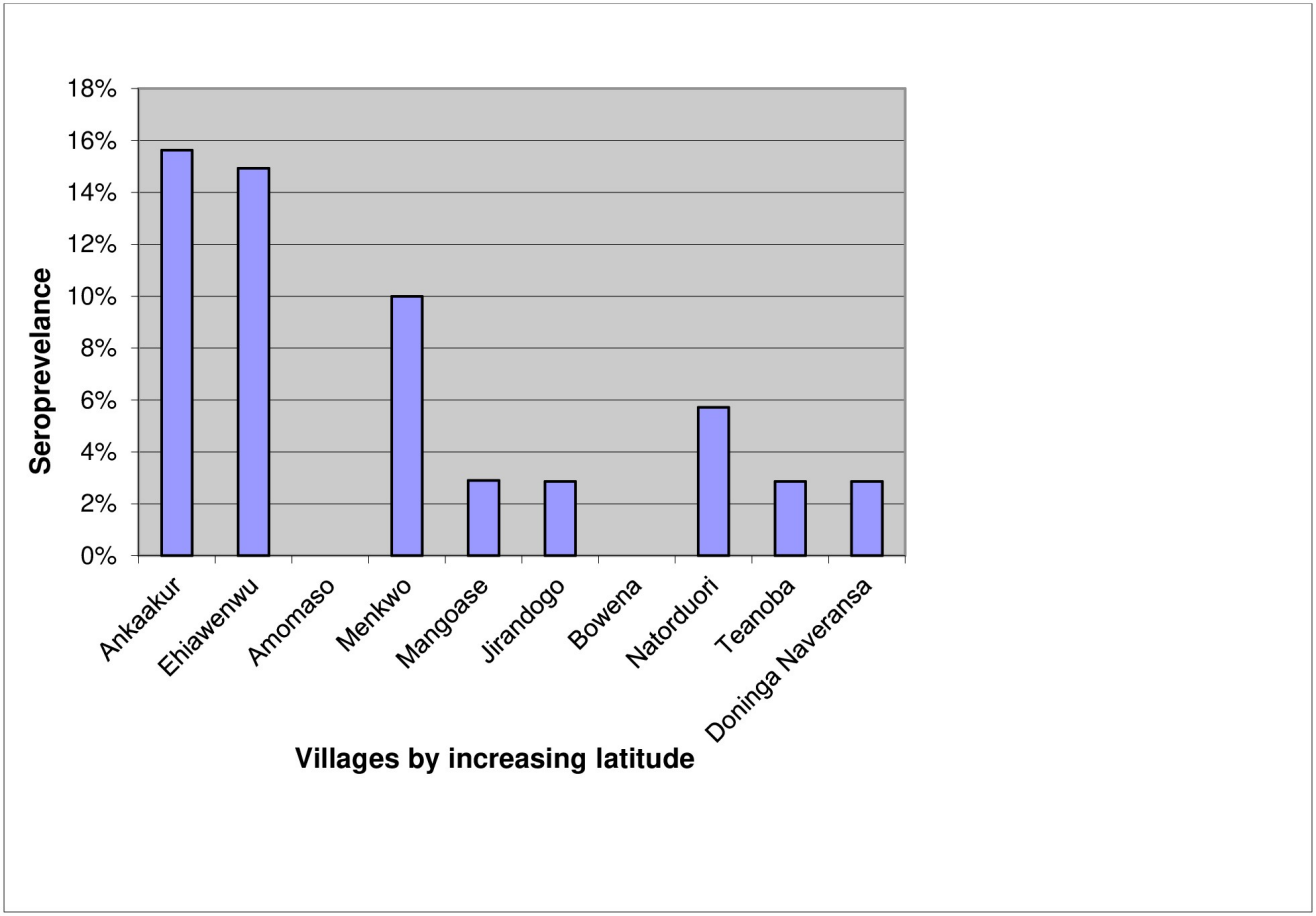
LASV seroprevalence by village.

**Fig 3:**
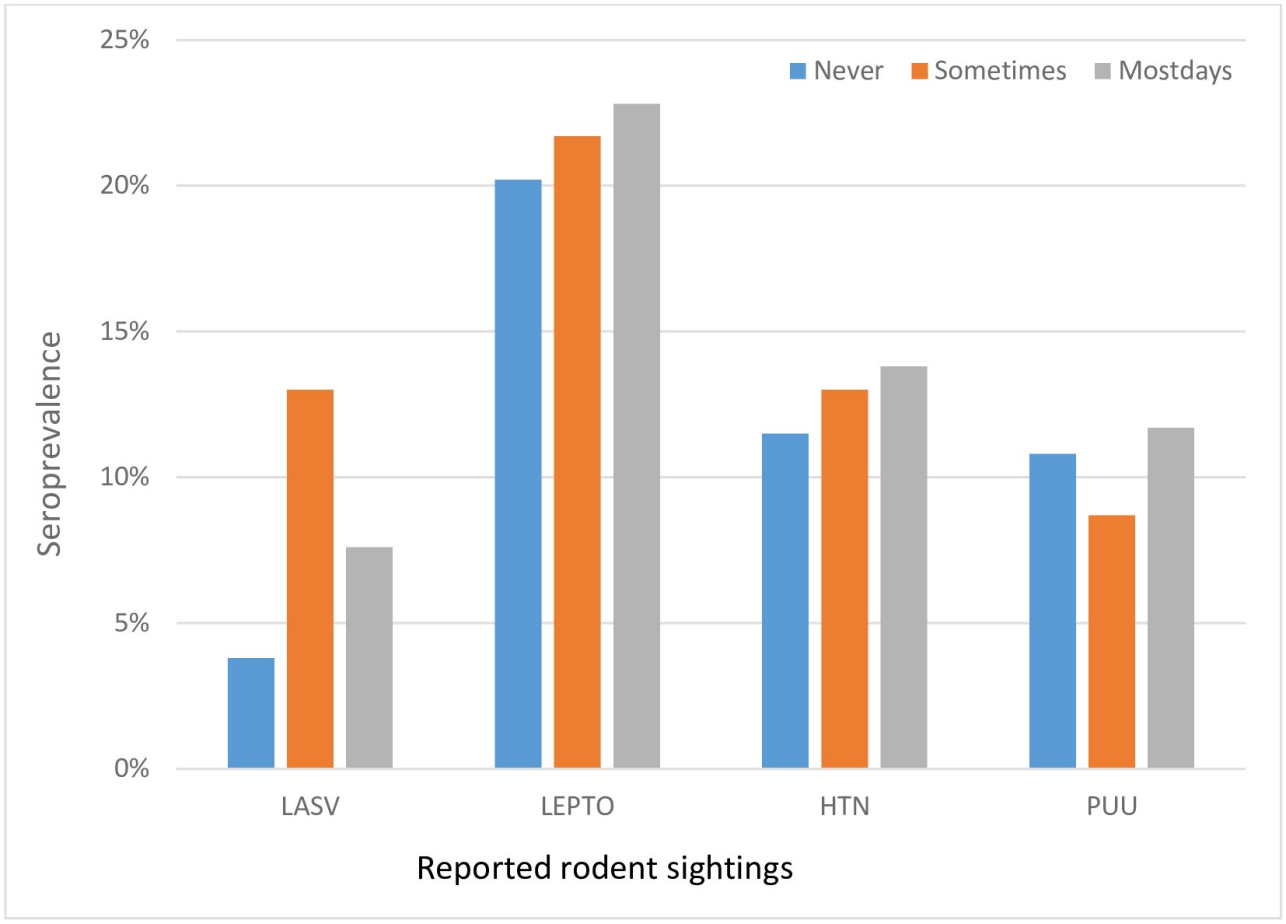
Seroprevalence and reported rodent sightings of four tested rodent-borne pathogens. (LASV-Lassa virus; PUU-Puumala serotype; HTN-Hantaan/Dobrava serotype; LEPTO-*Leptospira*).

Of 657 samples tested for LASV antibodies, 5.2% (95% CI 3.6–7.2) were seropositive by ELISA. Plaque reduction neutralization test performed on a subset of 15 samples tested negative. Individuals older than 65 years showed most ELISA seropositivity at 8.1% (95% CI 3.3–16.1) but there was no discernable age trend (Table 2). The slightly higher seroprevalence in males 9.1% (95% CI 4.2–10.2) compared to females 6.4% (95% CI 2.2–6.5) was not statistically significant (P = 0.11). Generally, there was no significant correlation between LASV antibody exposure and age (Chisq = 1.810, df = 1, P = 0.179). The seroprevalence in the three villages located in the moist deciduous forest region was 8.3%, while the seroprevalence in the seven villages in the more arid Guinea savanna woodland areas was 7.3% (P = 0.015). There was a significant effect of village on LASV antibodies (Chisq = 39.6, df = 9, P < 0.0001). Seropositivity by village varied from 0% (95% CI 0.0–5.1%) to 16% (95% CI 5.3–32.8) and was highest in the lower latitude villages (Fig 2). The seroprevalence of LASV antibodies for those living in aluminum roofed houses was 7% (95% CI 4.9–9.9) compared with 1% (95% CI 0.1–3.7) for those living under straw or other types of roofs. There was no significant interacting effect between roof type and wall type on LASV antibody status (Chisq = 0.16, df = 2, P = 0.923). LASV seroprevalence was also associated with the frequency of seeing rodents around the house. Paradoxically, those who reported never seeing rodents 17% (95% CI 2.8–33.6), were nearly four times more likely to have LASV antibodies than those who reported seeing rodents on most days 6% (95% CI 2.2–6.0) (Fig 3).

**Table 2:** Seroprevalence (%) of rodent-borne pathogens by age (yrs).

Age group	LASV IgG	P-Value	Puumala IgG	P-Value	Hantaan/Dobrava IgG	P-Value	Leptospira IgG	P-Value
18-25yrs	6.5	0.187	14.5	0.213	13.8	0.733	19.1	0.538
26-35yrs	2.0		7.2		10.5		21.9	
36-45yrs	3.7		13.1		9.5		25.0	
46-55yrs	6.9		11.1		13.9		20.0	
56-65yrs	7.3		9.1		16.4		27.9	
Above 65yrs	8.1		11.6		12.8		17.1	
Missing Age	0		0		0		66.7	
**Overall Seropositive**	**5.2**		**11.3**		**12.2**		**21.3**	

### Hantavirus (Dobrava and Puumala serotypes) immunoglobulin G

Tests for anti-Dobrava and Puumala antibodies were positive in 12.2% (95% CI 9.8–14.9) and 11.3% (95% CI 8.9–13.9) of samples, respectively. There was no significant correlation between both anti-hantavirus serotypes and age (Chisq = 0.991, df = 1, P = 0.320). However, ecological zone appeared to affect Dobrava virus prevalence (P = 0.029): Guinea savannah woodland 19 ± 2% and semi-deciduous forest 8 ± 2%. Additionally, the construction material used for a dwelling’s walls appears related to seropositivity for anti-Dobrava antibodies; 8% (95% CI 3.4–14.7) for brick or block, 13% (95% CI 9.9–15.8) for mud, and 23% (95% CI 8.9–43.7) for thatch. The relation between anti-Dobrava antibody exposure status and village (Akaike Information Criterion, AIC = 554) was better than for roof/wall type (AIC 575), which means that this effect of roof/wall type is likely to be due to the correlation between village and roof/wall type presence. There was no significant difference by housing wall type for Puumala antibodies (P = 0.237). There was a significant association between anti-Dobrava and Puumala antibodies, 7.1% (P < 0.0001). This correlation might be caused by ELISA cross-reactivity.

### *Leptospira* immunoglobulin G

A total of 140 samples showed anti-*Leptospira* antibodies corresponding to an overall seroprevelance of 21% (95% CI 18.2–24.6), the highest seropositivity rate of any of the rodent-borne disease tested. There was no statistically significant difference in *Leptospira* seropositivity and rodent observation frequency (Chisq = 0.021, df = 1, P = 0.885). There was however, a significant difference in *Leptospira* antibody prevalence between wall types (Chisq = 8.044, df = 2, P = 0.018). Seroprevalence of anti-*Leptospira* antibodies in males 22.5% and females 16.6% were significantly different (Chisq = 5.609, df = 1, P = 0.018).

### Positive sera for any rodent-borne disease

**Fig 4:**
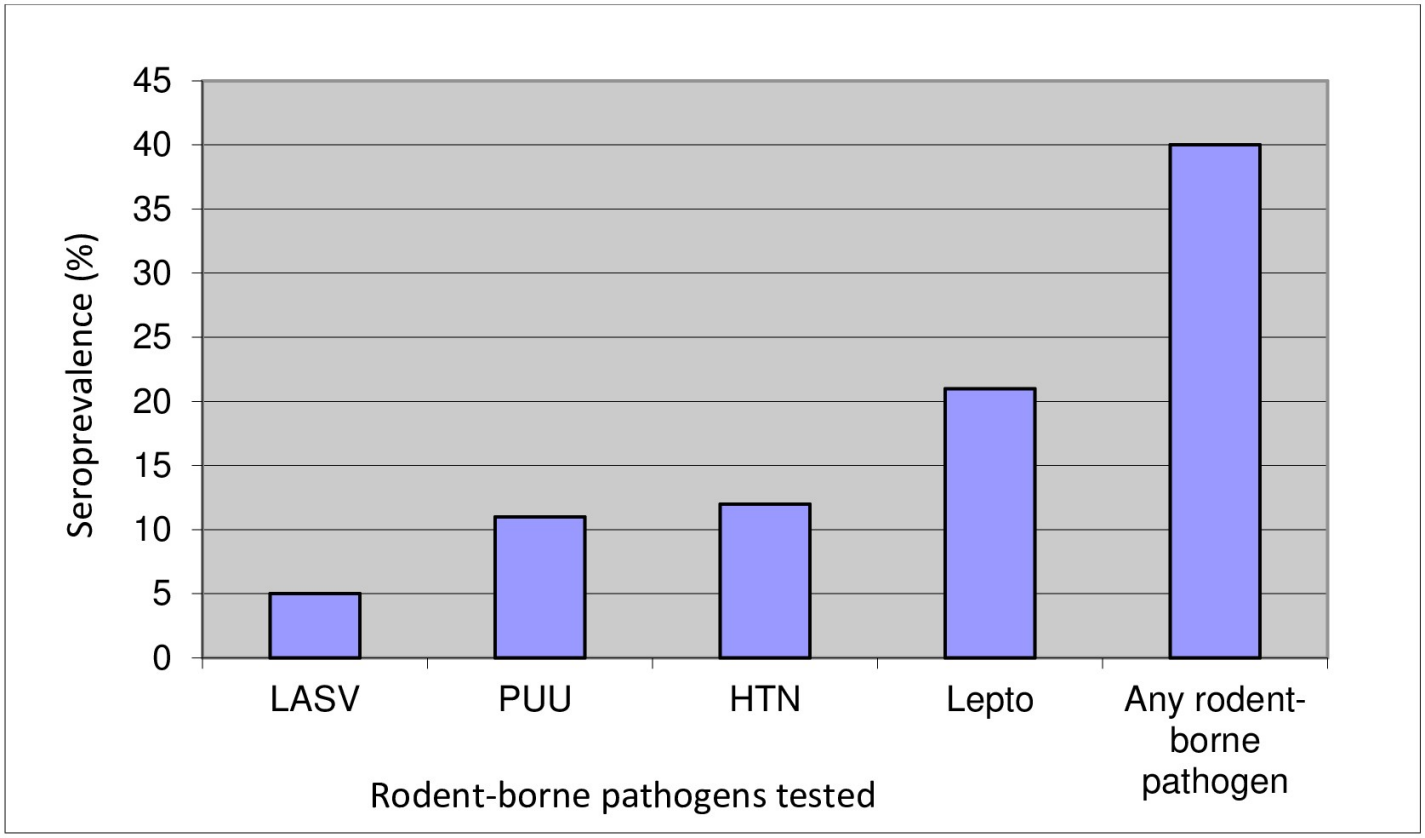
Seroprevalence of rodent-borne pathogens. (LASV-Lassa virus; PUU-Puumala serotype; HTN-Hantaan/Dobrava serotype; Lepto-*Leptospira*).

**Fig 5:**
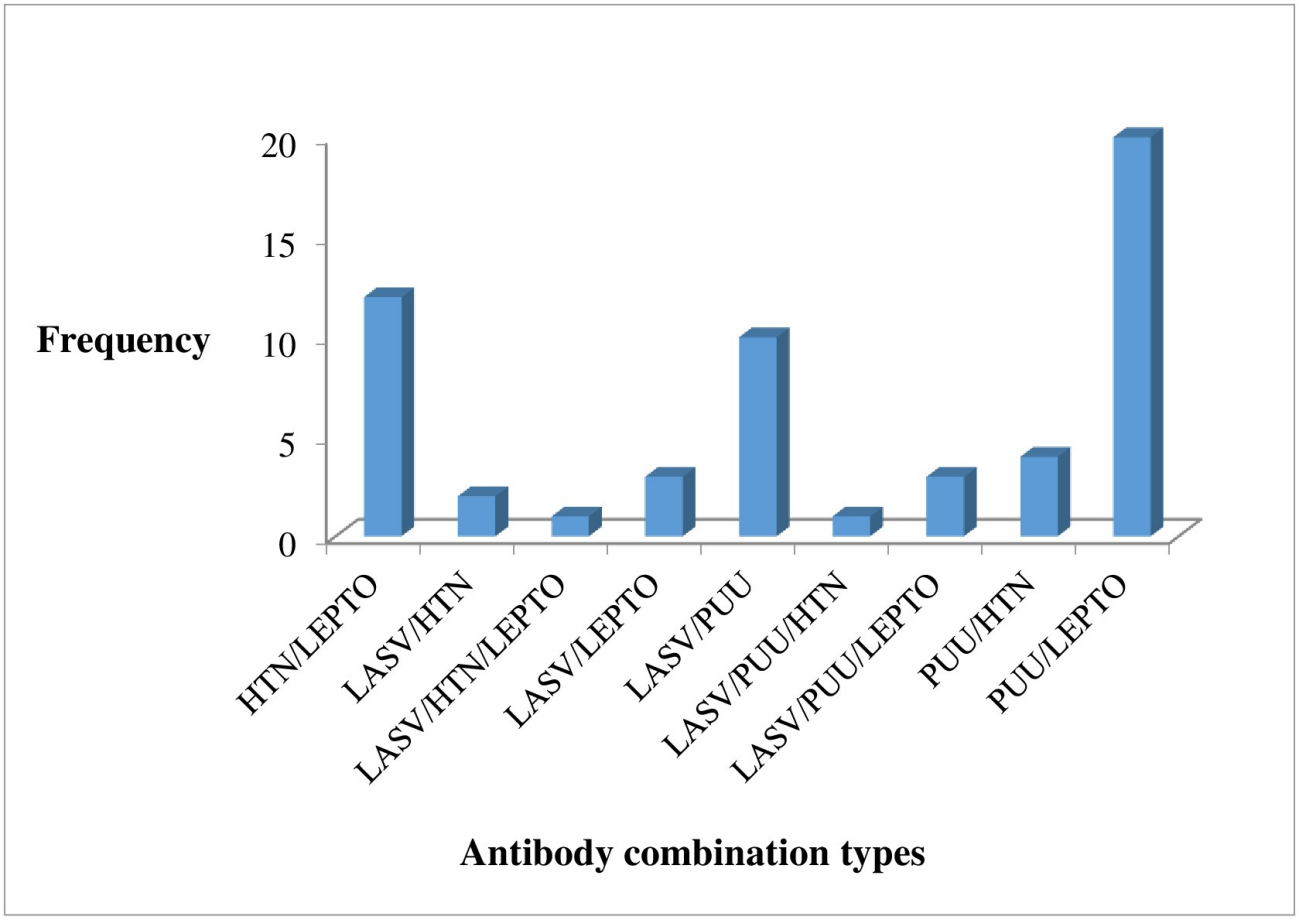
Multiple positive antibody combinations to rodent-borne pathogens.

Overall, 264 (40%) (95% CI 36.4–44.0) of individuals had antibodies to one or more of the four rodent-borne diseases tested, with leptospirosis being the most common (Fig 4). Fifty four of the 140 *Leptospira* positive samples, 39% (95% CI 30.5–47.2), were positive for another pathogen. Aside from the significant correlation between Dobrava and Puumala, the only significant correlation between pathogens was found for LASV and Puumala (Hantavirus serotype) [(P = 0.021; odds ratio = 2.35 (95% CI 1.23–4.40)]. Of the 34 positive LASV samples, 33% were also positive for Puumala antibodies, compared with 10% of the samples with negative LASV results (P < 0.001) (Fig 5). A significant positive association exists between rodent-borne pathogen seroprevalence and age (Chisq = 5.87, df = 1, P = 0.015) with an odds ratio = 1.011 (95% CI 1.0–1.0), suggesting that with every year the probability of being exposed increases by about 1.1%. With respect to reservoirs for the pathogens tested, there was no significant effect of reservoir ratio on LASV seroprevalence (Chisq = 2.679, df = 1, P = 0.102), or *Leptospira* (Chisq = 1.025, df = 1, P = 0.311), or Puumala (Chisq = 0.219, df = 1, P = 0.640), or Dobrava (Chisq = 0.073, df = 1, P = 0.788), or any of the four rodent-borne pathogens (Chisq = 0.227, df = 1, P = 0.634), or the number of antibody exposures (Chisq = 0.225, df = 1, P = 0.635).

## Discussion

While conducting this study, the high degree of human and rodent interaction was apparent in the communities. All were rural farming communities, generally without electricity or potable water. Food storage options were limited and opportunities for rodents to access stored food, as well as human living spaces, were plentiful. The rodents found in human dwellings had limited species diversity, but were found in high numbers [11]. Exposure to rodents is universal in these communities, and development of rodent-borne disease is likely heavily influenced by whether the species of commensal rodents inhabiting human spaces are viable carriers of human pathogens.

### Lassa virus infection

We previously reported that *Mastomys natalensis*, one of the rodent reservoirs of LASV, was the predominant commensal species in four of the ten study sites (namely, Bowena-28%, Menkwo-19%, Natorduori-16% and Doninga Naveransa-15%) though no LASV was detected in the captured rodents [11]. This study adds to the evidence that LASV may be circulating at low levels in rural Ghana. While 5% of human sera samples were positive by a LASV ELISA test, PRNT was negative. Possible reasons for LASV IgG antibodies detected by ELISA but not PRNT, include cross-reacting antibodies detected by ELISA but not PRNT, positive results of ELISA preceding or outlasting PRNT, or infection that does not result in neutralizing antibodies detected by PRNT. If due to cross-reacting antibodies, this could reflect infection with a similar pathogen, possibly another arenavirus related to LASV. The finding of two novel arenaviruses in Ghanaian rodents previously reported [11] supports this conclusion. It is not well known how long LASV antibodies remain detectable after infection, but the positive findings in samples from suspected risk communities suggest that LASV has circulated in human populations in rural Ghana.

We postulated that certain environmental or living conditions might allow better access to rodents or be more conducive to viral survival and transmission to humans. Both LASV and anti-Dobrava hantavirus antibody prevalence were significantly higher in the three southern semi-deciduous forest sites, where annual rainfall is higher than the Guinea savannah woodland sites. Annual rainfall has previously been reported as a risk factor for LASV survival outside the host [8]. In addition, residents who live under aluminum roofs were found to have significantly higher seropositivity for LASV. Aluminum roofs and higher annual rainfall could be associated with higher humidity and better viral survival. Given the ubiquitous presence of rodents, we do not suspect housing wall type to significantly affect rodent transmission rates, although this is also possible.

Surprisingly, subjects who reported seeing rodents frequently were less likely to have LASV antibodies. LASV is thought to be transmitted through ingestion of contaminated food or inhalation of contaminated dust, hantavirus through inhalation of contaminated dust, and leptospires through contact with contaminated soil or water. During our study, those who did not report seeing rodents may be less aware of the presence of rodents, and less likely to make corrective actions to protect food thus putting them at greater risk of exposure to rodents and pathogens they harbor. If this finding were duplicated in sites with known LASV transmission, it would suggest a role for public health education initiatives regarding rodent-borne disease awareness and secure food storage techniques.

### Infection with hantavirus

For hantavirus exposure, the observed IgG seroprevalence of 11.75% is similar to that previously reported in the forest of Guinea (12.2%; [29]) and Gabon (8%; [30]), but higher than in the central African countries (4%; [13]). Confirmatory tests in the Guinea study resulted in a significantly lower seroprevalence rate of 1.2%. No confirmatory tests were done in our study. Nonetheless, in this initial assessment from Ghana, our goal was to provide an early estimate of the potential burden of disease exposure. Further studies are warranted to better determine the extent of hantavirus disease in Ghana.

### *Leptospira* infection

Leptospirosis has not been widely studied in Africa and clinical diagnosis alone is difficult [31]. About 21% of the subjects had antibodies to *Leptospira* species via an enzyme immnunoassay. The overall seroprevalence in this survey was lower than the 33% rate demonstrated by Hogerzeil and others in 1986 within the humid forest areas of Ghana [32]. Comparing the seroprevalence of the ecological zone in the 1986 study with this survey, we observed a 29% seroprevalence of *Leptospira* exposure. Other previous studies conducted in West Africa reported 18% among volunteers in Nigeria [33]. Studies in acute febrile patients will be necessary to better determine the burden of disease in Ghana.

This study has several limitations. Our primary goal was to detect evidence of LASV if it is indeed circulating at low levels in humans in Ghana. The volunteers in each village were a convenience sample of willing volunteers and not randomly selected, introducing potential bias. In addition, confirmatory LASV serology was done using Josiah strain virus, which could underestimate seroprevalence for another, unknown strain of LASV in circulation in Ghana. No confirmatory testing was done for the other pathogens besides LASV, and results are subject to the limitations of ELISA-based testing with commercial kits. Sites were chosen to provide broad geographical coverage of Ghana and include areas identified through modeling as having increased risk for Lassa fever. However, limited areas of transmission could have been missed within these areas, or in areas not currently suspected of having risk. This study is also limited to one point in time (sampling was done once in each of the villages) and periodic circulation is also possible, though negative serology makes that possibility less likely.

## Conclusion

This study found that 40% of residents in selected rural farming communities in Ghana have antibodies to one or more rodent-borne diseases. Leptospirosis was the most prevalent of the rodent-borne diseases we tested, though LASV, Dobrava and Puumala viruses were also present in at least 5% of the participants. We found high seroprevelance for rodent-borne diseases, including LASV in Ghana, which neighbors countries with recent LASV outbreaks pointing to the need for continued surveillance and study.

## Supporting information

S1 File Questionnaire. (PDF)

S1 Table Demographic information of villages sampled. (DOCX)
