# Correction: Rodent-borne infections in rural Ghanaian farming communities

**DOI:** 10.1371/journal.pone.0218271

**Published:** 2019-06-06

**Authors:** Shirley C. Nimo-Paintsil, Elisabeth Fichet-Calvet, Benny Borremans, Andrew G. Letizia, Emad Mohareb, Joseph H. K. Bonney, Kwasi Obiri-Danso, William K. Ampofo, Randal J. Schoepp, Karl C. Kronmann

Fig 3 is incorrect. The authors have provided a corrected version here.

**Fig 3:**
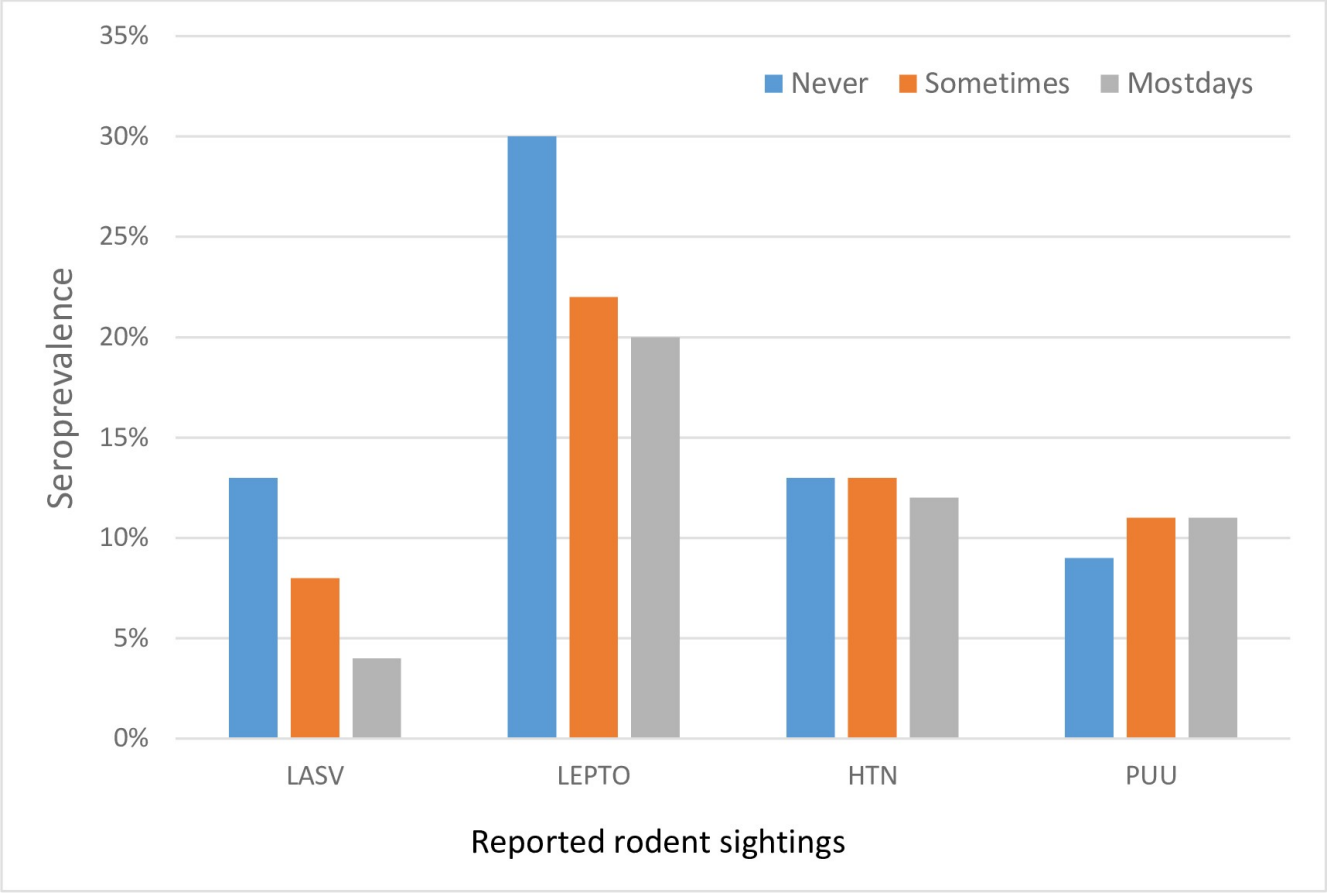
Seroprevalence and reported rodent sightings of four tested rodent-borne pathogens. (LASV-Lassa virus; PUU-Puumala serotype; HTN-Hantaan/Dobrava serotype; LEPTO-*Leptospira*).
