# Supplementary material for: Rodent-borne infections in rural Ghanaian farming communities
Source: PLoS One. 2019 Apr 24;14(4):e0215224. doi: 10.1371/journal.pone.0215224 (PMC6481813; doi:10.1371/journal.pone.0215224)
Supplement: S1 File — (PDF) [file pone.0215224.s001.pdf]

1. Case ID No.: **Date:** ___/___/_____

2. ________________ ________________ ____________________
Village **GPS Coordinates** **Region**

Demographics

3. Name of Participant: ______________________ **4. Date of Birth:** ____/____/_____ **Age:** _____ **years**

5. Sex: ☐Female ☐Male

6. Residence **Wall Type:** ☐ Mud ☐ Block ☐ Other_______ **Roof:** ☐ Aluminum ☐ Straw ☐ Other _______

Do you have: **Indoor plumbing?** ☐ Yes ☐ No **Electricity?** ☐ Yes ☐ No

7. Number of persons living in your house: _____

8. Where is food stored? ☐ Separate building ☐ Kitchen ☐ Room in house ☐ Other _____

9. Occupation: ☐**Business** ☐**Farmer (Circle all applicable: Rice Maize Livestock other __________)**
☐ **Other (List)**_______________________

10. Does your family own any of the following?

☐None	☐Swine	☐Pigeons	☐Dogs
☐Sheep	☐Donkeys	☐Ducks	☐Bats
☐Goats	☐Horses	☐Other Birds	☐Grasscutters
☐Cattle	☐Chickens	☐Cats	☐Rabbits

11. Do you drink fresh milk? ☐ Yes ☐ No **If yes, what type?** ☐ Cow ☐ Goat

12. Have you been around animals giving birth (live or dead)? ☐ Yes ☐ No

13. Have you seen rodents around your house? ☐ Most days ☐ Sometimes ☐ Never

14. Have you been bitten by a tick before? ☐ Yes ☐ No

Health

15. How many times have you had malaria or fever this year? ________

16. Have you ever been admitted to the hospital? ☐ Yes ☐ No **What was diagnosis?** ______________________

17. Do you have any problems with hearing? ☐ Yes ☐ No

18. Do you sleep under a mosquito net? ☐ Yes ☐ No

Person Completing Form **Name:** ________________________ **Initials** _______

NMIMR INSTITUTIONAL REVIEW
VALID UNTIL
2 2 AUG 2012
APPROVED DOCUM[illegible]

*If lab specimen is collected, complete the following information and send a copy of this form to the lab with the specimen.

19. Date of specimen collection: ____/____/________

For the Laboratory Use Only

20. Date specimen received by lab: ____/____/______ **21. Specimen Condition:** ☐Adequate ☐Not adequate ______________ (Reason)

22. Results: Check (X) below as appropriate:

Disease/ Condition	Type of antibody	Results (+=positive) (-=negative)	Titer (if done)	Type of test (e.g. ELISA)
Lassa	IgG	☐+ ☐ -	________	
Leptospirosis	IgG	☐+ ☐ -	________	
Melioidosis	IgG	☐+ ☐ -	________	
Brucellosis	IgG	☐+ ☐ -	________	
Q Fever	IgG	☐+ ☐ -	________	

COMMENTS:

NMIMR INSTITUTIONAL REVIEW BOARD
VALID UNTIL
2 2 AUG 2012
APPROVED DOCUMENT
