# Supplementary material for: Rodent-borne infections in rural Ghanaian farming communities
Source: PLoS One. 2019 Apr 24;14(4):e0215224. doi: 10.1371/journal.pone.0215224 (PMC6481813; doi:10.1371/journal.pone.0215224)
Supplement: S1 Table — (DOCX) [file pone.0215224.s002.docx]

**S1 Table: Demographic information of villages sampled**

| **VILLAGES** | **AMOM**  **N (%)** | **ANKA**  **N (%)** | **BOWE**  **N (%)** | **DONA**  **N (%)** | **EHIA**  **N (%)** | **JIRA**  **N (%)** | **MANG**  **N (%)** | **MONK**  **N (%)** | **NATO**  **N (%)** | **TEAN**  **N (%)** | **P-Value** |
| --- | --- | --- | --- | --- | --- | --- | --- | --- | --- | --- | --- |
| **GENDER** | | | | | | | | | | | 0.242 |
| Male | 27(39) | 11(36) | 29(41) | 25(36) | 36(54) | 32(46) | 29(41) | 38(54) | 33(47) | 37(53) |  |
| Female | 43(61) | 20(65) | 41(59) | 44(63) | 31(46) | 37(54) | 40(57) | 32(46) | 35(50) | 33(47) |  |
| Missing data | 0(0) | 0(0) | 0(0) | 1(1) | 0(0) | 0(0) | 1(1) | 0(0) | 2(3) | 0(0) |  |
| **AGE** | | | | | | | | | | | |
| 18-25yrs | 12(17) | 10(33) | 12(17) | 14(20) | 7(11) | 18(27) | 18(26) | 25(36) | 15(21) | 22(31) | <0.001 |
| 26-35yrs | 20(29) | 4(13) | 19(27) | 19(27) | 5(8) | 17(25) | 14(20) | 14(20) | 15(21) | 25(36) |  |
| 36-45yrs | 12(17) | 5(17) | 10(14) | 9 (13) | 14(21) | 17(25) | 25(36) | 12(17) | 18(25) | 14(20) |  |
| 46-55yrs | 17 (24) | 2 (7) | 11(16) | 10(14) | 8(12) | 6 (9) | 3 (4) | 7 (10) | 3 (4) | 5(7) |  |
| 56-65yrs | 6 (9) | 3 (10) | 7 (10) | 8( 11) | 8 (12) | 4 (6) | 4 (6) | 6 (9) | 6 (9) | 3(4) |  |
| Above 65yrs | 3(4) | 6(20) | 11(16) | 10(14) | 25(37) | 6(9) | 5(7) | 6(9) | 13(19) | 1(1) |  |
| Missing age | 0(0) | 1(3) | 0(0) | 0(0) | 0(0) | 1 (1) | 1(1) | 0(0) | 0(0) | 0(0) |  |

| **VILLAGES** | **AMOM**  **N (%)** | **ANKA**  **N (%)** | **BOWE**  **N (%)** | **DONA**  **N (%)** | **EHIA**  **N (%)** | **JIRA**  **N (%)** | **MANG**  **N (%)** | **MONK**  **N (%)** | **NATO**  **N (%)** | **TEAN**  **N (%)** | **P-Value** |
| --- | --- | --- | --- | --- | --- | --- | --- | --- | --- | --- | --- |
| **HOUSING, OCCUPANCY** | | | | | | | | | | | <0.001 |
| 1-5 persons | 30(43) | 13(42) | 15(21) | 10(14) | 26(39) | 2(3) | 23(33) | 11(16) | 20(29) | 2(3) |  |
| 6-10 persons | 37(53) | 12(39) | 29(41) | 15(21) | 34(51) | 27(39) | 39(56) | 47(67) | 31(44) | 16(23) |  |
| Greater than 10 persons | 3(4) | 6(19) | 23(33) | 44(63) | 7(11) | 40(58) | 7(10) | 11(16) | 16(23) | 52(74) |  |
| Missing data | 0(0) | 0(0) | 3(4) | 1(1) | 0(0) | 0(0) | 1(1) | 1(1) | 3(4) | 0(0) |  |
| **HOUSING, WALL TYPE** | | | | | | | | | | | <0.001 |
| Block or brick | 7(10) | 0(0) | 50(71) | 11(16) | 20(30) | 1(2) | 3(4) | 6(9) | 5(7) | 0(0) |  |
| Mud | 61(87) | 29(94) | 19(27) | 59(84) | 45(67) | 68(99) | 42(60) | 63(90) | 65(93) | 70(100) |  |
| Thatch | 0(0) | 2(7) | 0(0) | 0(0) | 0(0) | 0(0) | 24(34) | 0(0) | 0(0) | 0(0) |  |
| Missing data | 2(3) | 0(0) | 1(1) | 0(0) | 2(3) | 0(0) | 1(1) | 1(1) | 0(0) | 0(0) |  |
| **HOUSING, ROOF TYPE** | | | | | | | | | | | <0.001 |
| Aluminium | 69 (99) | 25 (81) | 7 (10) | 32 (46) | 66 (99) | 62 (90) | 24 (34) | 49 (70) | 66 (94) | 51 (73) |  |
| Straw, Thatch or Bamboo | 1(1) | 6 (19) | 61 (87) | 38 (54) | 1 (1) | 7 (10) | 40 (57) | 18 (26) | 3 (4) | 19 (27) |  |
| Missing data | 0 (0) | 0 (0) | 0 (0) | 0 (0) | 0 (0) | 0 (0) | 6 (9) | 3 (4) | 1(1) | 0 (0) |  |
| **VILLAGES** | **AMOM**  **N (%)** | **ANKA**  **N (%)** | **BOWE**  **N (%)** | **DONA**  **N (%)** | **EHIA**  **N (%)** | **JIRA**  **N (%)** | **MANG**  **N (%)** | **MONK**  **N (%)** | **NATO**  **N (%)** | **TEAN**  **N (%)** | **P-Value** |
| **HOUSING, INDOOR PLUMBING** | | | | | | | | | | | <0.001 |
| YES | 0 (0) | 0 (0) | 0 (0) | 0 (0) | 0 (0) | 0 (0) | 35 (50) | 1 (1) | 31(44) | 0 (0) |  |
| NO | 70 (100) | 31(100) | 70 (100) | 70 (100) | 67 (100) | 69 (100) | 33 (47) | 66 (94) | 37 (53) | 70 (100) |  |
| MISSING DATA | 0 (0) | 0 (0) | 0 (0) | 0 (0) | 0 (0) | 0 (0) | 2 (3) | 3 (4) | 2 (3) | 0 (0) |  |
| **HOUSING, ELECTRICITY** | | | | | | | | | | | <0.001 |
| Yes | 0 (0) | 0(0) | 0 (0) | 4 (6) | 0(0) | 34 (49) | 0 (0) | 1 (1) | 0(0) | 0 (0) |  |
| No | 70 (100) | 31 (100) | 70 (1000 | 66(94) | 67 (100) | 35 (51) | 64 (91) | 67 (96) | 62 (89) | 70 (100) |  |
| Missing data | 0 (0) | 0 (0) | 0 (0) | 0 (0) | 0 (0) | 0 (0) | 6 (9) | 2 (3) | 8 (11) | 0 (0) |  |
| **RODENT OBSERVATION** | | | | | | | | | | | <0.001 |
| Most days | 60 (88) | 29 (97) | 60 (86)) | 32 (46) | 28 (42)) | 67 (99) | 38 (55) | 36 (52) | 24 (35) | 54 (77) |  |
| Sometimes | 8 (12) | 1 (3) | 10 (14) | 33 (48) | 37 (55) | 1 (1) | 31(45) | 25 (36) | 36 (53) | 15 (21) |  |
| Never | 0 (0) | 0 (0) | 0 (0) | 4 (6) | 2(3) | 0 (0) | 0 (0) | 8 (12) | 8 (12) | 1(1) |  |
| Missing data | 2 (3) | 1 (3) | 0 (0) | 1 (1) | 0 (0) | 1 (1) | 1(1) | 1(1) | 2(3) | 0(0) |  |
